# Somato-Dendritic Regulation of Raphe Serotonin Neurons; A Key to Antidepressant Action

**DOI:** 10.3389/fnins.2018.00982

**Published:** 2018-12-20

**Authors:** Emily Quentin, Arnauld Belmer, Luc Maroteaux

**Affiliations:** ^1^INSERM UMR-S 839, Institut du Fer à Moulin, Paris, France; ^2^Sorbonne Universités, UPMC University Paris 6, Paris, France; ^3^Institut du Fer à Moulin, Paris, France

**Keywords:** serotonin receptors, somatodendritic release, volume transmission, antidepressants, autoreceptors

## Abstract

Several lines of evidence implicate serotonin (5-hydroxytryptamine, 5-HT)in regulating personality traits and mood control. Serotonergic neurons are classically thought to be tonic regular-firing, “clock-like” neurons. Neurotransmission by serotonin is tightly regulated by the serotonin transporter (SERT) and by autoreceptors (serotonin receptors expressed by serotonin neurons) through negative feedback inhibition at the cell bodies and dendrites (5-HT_1A_ receptors) of the dorsal raphe nuclei or at the axon terminals (5-HT_1B_ receptors). In dorsal raphe neurons, the release of serotonin from vesicles in the soma, dendrites, and/or axonal varicosities is independent of classical synapses and can be induced by neuron depolarization, by the stimulation of L-type calcium channels, by activation of glutamatergic receptors, and/or by activation of 5-HT_2_ receptors. The resulting serotonin release displays a slow kinetic and a large diffusion. This process called volume transmission may ultimately affect the rate of discharge of serotonergic neurons, and their tonic activity. The therapeutic effects induced by serotonin-selective reuptake inhibitor (SSRI) antidepressants are initially triggered by blocking SERT but rely on consequences of chronic exposure, i.e., a selective desensitization of somatodendritic 5-HT_1A_ autoreceptors. Agonist stimulation of 5-HT_2B_ receptors mimicked behavioral and neurogenic SSRI actions, and increased extracellular serotonin in dorsal raphe. By contrast, a lack of effects of SSRIs was observed in the absence of 5-HT_2B_ receptors (knockout-KO), even restricted to serotonergic neurons (*Htr2b^5-HTKO^* mice). The absence of 5-HT_2B_ receptors in serotonergic neurons is associated with a higher 5-HT_1A_-autoreceptor reactivity and thus a lower firing activity of these neurons. In agreement, mice with overexpression of 5-HT_1A_ autoreceptor show decreased neuronal activity and increased depression-like behavior that is resistant to SSRI treatment. We propose thus that the serotonergic tone results from the opposite control exerted by somatodendritic (Gi-coupled) 5-HT_1A_ and (Gq-coupled) 5-HT_2B_ receptors on dorsal raphe neurons. Therefore, 5-HT_2B_ receptors may contribute to SSRI therapeutic effects by their positive regulation of adult raphe serotonergic neurons. Deciphering the molecular mechanism controlling extrasynaptic release of serotonin, and how autoreceptors interact in regulating the tonic activity of serotonergic neurons, is critical to fully understand the therapeutic effect of SSRIs.

## Introduction

In any given year, nearly 40% of the population in European countries is affected, directly or indirectly, by mental illness (Insel and Sahakian, 2012). Mental illness or psychiatric diseases are heterogeneous pathologies and much effort remains necessary to improve diagnosis and therapies. For example, 30–40% of patients with major depression do not respond to current treatments, which suggests that ontogeny of the disease may vary among individuals, and that novel pathways and therapeutic targets have to be identified. Serotonin (5-hydroxytryptamine, 5-HT) is implicated in the processing of perception, emotion, and cognitions and has been involved in various psychiatric disorders (Krishnan and Nestler, 2008). Several lines of evidence implicate serotonin in regulating personality traits and mood control. Indeed, serotonin has also been implicated in the etiology of several mood disorders, including autism spectrum disorders (ASD), major depressive disorder (MDD), schizophrenia or bipolar disorder (BD) (Vadodaria et al., 2018). Accordingly, a growing interest in understanding the molecular and cellular effect of many therapeutic compounds has emerged: serotonin transporter (SERT) is the main target of serotonin selective reuptake inhibitor (SSRI) antidepressants, and 5-HT_2_ receptors are targets of atypical antipsychotics.

Variations in serotonin levels may affect mood and motivation but functions of endogenous serotonin remain controversial. It has been recently suggested that serotonin enables organisms to adapt to dynamic environments by controlling neuronal plasticity and behavior (Matias et al., 2017). Therefore, the clinical benefits of improving serotonin function would stem from facilitating adaptive changes to negative affects rather than positively modulating the emotional states (Branchi, 2011). Serotonergic neurons are classically thought to display regular tonic firing, or “clock-like,” neurons (Jacobs and Azmitia, 1992), whereas phasic firing in bursts is associated with specific behaviors. Phasic and tonic firing of serotonergic neurons have also been proposed to have opposite functions. However, the respective contribution of serotonergic mode of firing to behavior remains unclear. Tonic firing of serotonin neuron population activity seems related to the extra-synaptic tonic serotonin levels and burst firing to the rapid, high-amplitude, and intra-synaptic phasic serotonin release.

However, how the positive modulation of serotonin tone translates into raised mood or decreased anxiety is not yet understood and the precise relationship between certain behaviors and brain serotonin levels remains unclear. For instance, anxiolysis as a result of reducing brain serotonin is well established, suggesting that serotonin increases anxiety. However, anxiety is often paired with depression, which is classically associated with low serotonin levels (Jennings et al., 2010). Also, SSRIs are effective in treating both disorders, but only in a fraction of patients. Therefore, the precise relationship between serotonin levels and behavior is still to be established. Studies to date have not provided a sufficiently detailed understanding of how tonic serotonin neuron activity can be related to serotonin levels. In this review, we will summarize the known molecular mechanisms controlling tonic release of serotonin, in which autoreceptors (serotonin receptors expressed by serotonin neurons) and SERT participate in regulating the excitability of serotonergic neurons. An understanding of the detailed dynamics of serotonin dendritic release might clarify how serotonin governs behavior, which is critical to fully understand the therapeutic effect of SSRIs.

## The Two Modes of Monoamine and Serotonin Transmission

In the brain, neuronal communication is mediated by two major modes of chemical transmission. In the presynaptic terminal, neurotransmitters are released rapidly and locally, and signal to post-synaptic partners for synaptic transmission. In “non-synaptic” transmission, by contrast, neuromodulators diffuse over a large area to stimulate surrounding cells including glial cells (Agnati et al., 1995). In fast neurotransmission, the active zone, which is formed by defined and ordered protein network and docks synaptic vesicles, releases neurotransmitters in millisecond timing. By enhancing their release probability, this neurotransmission allows ordered vesicles to fuse in front of post-synaptic neurotransmitter receptors (Südhof, 2012). The non-synaptic mode of transmission does not take place between two pre- and post-synaptic elements as described above, and neuromodulators are released in a pseudo-open space. Thus, non-synaptic transmission is defined as “volume transmission” (Agnati et al., 1995; Zoli et al., 1999) and lasts for seconds. Precise organization of secretion is not necessary for volume transmission. This signal, which is slow and diffuses in a space larger than the synaptic cleft, involves a low concentration of neurotransmitters.

Monoamine (including serotonin) release has been subdivided into tonic and phasic modes. Tonic release controls the large variation in extracellular monoamine through basal and non-synchronous firing of neurons; by contrast, in phasic release, synchronized burst firing results in a fast, large, and transient neuromodulator increase (Grace, 2016). These neurochemical findings correspond to different neuronal activities. For example, the tonic activity of serotonin neurons can be related to extra-synaptic serotonin-containing vesicle release; the burst firing can be related to the rapid, high-amplitude, intra-synaptic phasic serotonin-containing vesicle release. Tonic firing is characterized by low frequency (0.1–3 Hz), and is classically defined as having clock-like, pace-maker regularity. Phasic firing characterized with burst of higher firing rates (up to 17 Hz) has indeed been reported in serotonin neurons (Allers and Sharp, 2003; Kocsis et al., 2006; Hajós et al., 2007). The precise control of neuronal activity that differentiates these two modes of release is not yet well understood.

The existence of serotonin volume transmission has been supported by several observations, (1) the distribution of serotonergic receptors and transporter not facing post-synaptic densities suggests that they detect serotonin released extrasynaptically (Ridet et al., 1994; Bunin and Wightman, 1999); this is notably the case for the 5-HT_1A_ receptor, which is known to play an autoreceptor function in the dorsal raphe (Kia et al., 1996; Riad et al., 2000); (2) serotonin- and vesicular transporter (VMAT2)-positive vesicles are found not only in axonal varicosities, but also in the soma and dendrites; these VMAT2-positive vesicles are located independently of post-synaptic elements (Chazal and Ralston, 1987; Descarries and Mechawar, 2000), suggesting that non-synaptic vesicular storage and release can also occur in the somatodendritic compartment; (3) finally, it has been shown that similar amount of serotonin can be found at the somatic or dendritic level compared to axonal terminals (Bruns et al., 2000; Kaushalya et al., 2008b); in addition, extracellular concentrations of serotonin can increase in response to single stimulation pulses (Bunin and Wightman, 1999). Extrasynaptic release mechanisms likely occur by regulated exocytosis of vesicles (Trueta and De-Miguel, 2012) leading a widespread release in the extracellular space.

In axons, serotonin can be released from presynaptic terminals, but also from extra-synaptic sites (varicosities). In axonal varicosities, in dendrites and in soma, serotonin is released via volume transmission. The tonic activity of serotonin neurons being related to extra-synaptic serotonin release is likely to use volume transmission. However, the vesicular release machinery for this mode of transmission may be different from that used for synaptic transmission.

## Vesicular Complexes Involved in Serotonin Release by Volume Transmission

**FIGURE 1:**
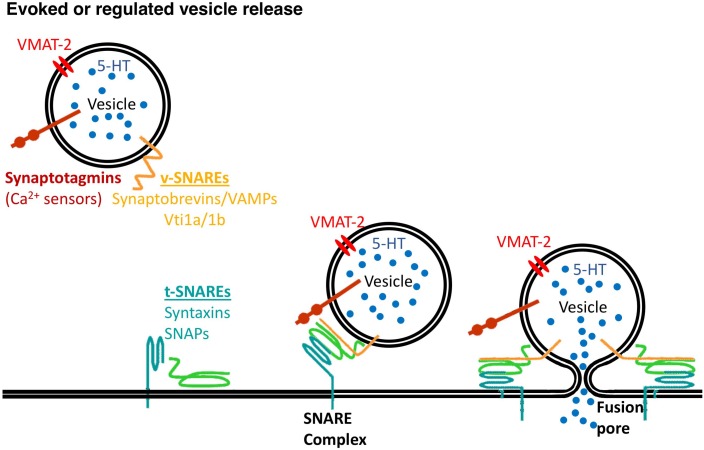
Vesicle release needs the SNARE proteins. v-SNARE proteins, synaptobrevins/VAMPs, Vti1a/1b and t-SNARE proteins, syntaxins and synaptosomal-associated proteins (SNAPs) mediate synaptic vesicles fusion to the plasma membrane with a contribution of calcium sensors, synaptotagmins.

Members of the family of soluble N-ethylmaleimide-sensitive fusion protein-attachment protein receptors (SNAREs) are involved in intracellular vesicular trafficking. The association of SNARE proteins expressed by interacting membranes triggers exocytosis by forming complexes through four coiled-coil SNARE motifs (Jahn and Scheller, 2006). Evoked synaptic vesicle release needs the canonical SNARE proteins, including the vesicle-associated SNAREs (v-SNAREs) synaptobrevin 2 that interacts with target membrane SNAREs (t-SNAREs) syntaxin 1 and SNAP-25 that are required for vesicle fusion (Figure 1 and Table 1).

**Table 1:** Vesicles-associated molecules and mRNA expression in serotonergic neurons.

Molecule	Type	Expression in 5-HT Neurons^D^
**Vesicular SNAREs (v-SNAREs)**^B,R^
Synaptobrevin 1/VAMP1	NC	++
Synaptobrevin 2/VAMP2	C	++++
Vamp3	NC	+
Vamp4	NC	++
Vamp7	NC	+
Vti1a	NC	+
Vti1b	NC	++
**Target membrane SNAREs (t-SNAREs)**^B,R^
Syntaxin Stx1a	C	+
Stx1b	C	+++
Stx2	NC	+
Stx3	NC	+
Stx4a	NC	++
Stx5a	NC	+
Stx6	NC	+
Stx7	NC	++
Stx8	NC	+
Stx12	NC	+++
Stx16	NC	++
Stx17	NC	+
Stx18	NC	+
SNAP-25	C	+++++
SNAP-29	NC	+
**Calcium sensors**^B,R^
Synaptotagmin Syt1		++++
Syt2		+
Syt3		+
Syt4		+++
Syt5		++
Syt6		+
Syt7		+
Syt9		++
Syt11		+++
Syt12		+
Syt13		+++
Syt16		+
Syt17		+


*C, Canonical SNAREs; NC, non-canonical SNAREs; data are from ^*D*^(Okaty et al., 2015), ^*R*^(Ramirez and Kavalali, 2012), and ^*B*^(Burré, 2007).*

Volume transmission likely involves a particular vesicular machinery. Vesicular transporters traffic to synaptic vesicles as well as large dense core vesicles (Fei et al., 2008). It has been shown that, in transfected neurons, VMAT-2 is spontaneously targeted to the regulated secretory pathway and is sufficient to drive regulated exocytotic release of monoamine (Li et al., 2005). In midbrain, it has been recently reported that axons of dopamine neurons contain non-synaptic release sites (varicosities) that are required for action potential-triggered dopamine release in 30% of dopamine vesicle clusters, leading to the conclusion that a large proportion dopamine varicosities release dopamine independently of action potentials and thus use a different exocytotic release machinery (Liu et al., 2018).

If synaptic transmission mechanisms are well described, volume transmission mechanisms remain to be precisely investigated. Vesicles exocytosis might use similar machinery to the evoked transmitter-release exocytosis of neurons and neurosecretory cells. Regulated release likely uses the non-canonical SNARE proteins, present in serotonergic neurons (Okaty et al., 2015) and listed in Table 1 including VAMP4, VAMP7 (Raingo et al., 2012; Bal et al., 2013), Vti1a or Vti1b (Kunwar et al., 2011; Ramirez et al., 2012), see for reviews (Burré, 2007; Ramirez and Kavalali, 2012). Whether volume transmission uses a mechanism more closely related to regulated vesicular release rather than classical synaptic release has to be further investigated.

## Models of Somatodendritic Serotonin Release

The mechanisms of non-synaptic serotonin release are difficult to study in physiological situations. Therefore, only few models of non-synaptic serotonin release have been described. Serotonin can be non-synaptically released at somatodendritic, pure somatic and/or pure dendritic compartments, with different control mechanisms (de Kock et al., 2006; Kaushalya et al., 2008a; Colgan et al., 2009; Leon-Pinzon et al., 2014).

### In Leeches

**FIGURE 2:**
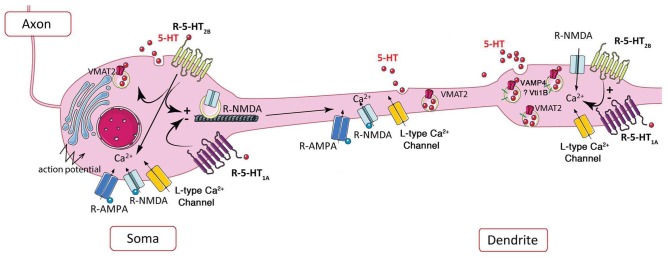
Schematic representation of the potential role of 5-HT_2B_ receptor in extrasynaptic release of serotonin. Left, in soma, the somatic release of serotonin depends on AMPA and NMDA receptors, L-type calcium channels, and action potentials, 5-HT_1A_ and potentially 5-HT_2B_ receptor via its coupling to PKC (Trueta et al., 2003, 2004; de Kock et al., 2006; Colgan et al., 2012; Leon-Pinzon et al., 2014). Activation of 5-HT_1A_ and 5-HT_2B_ receptors may decrease or increase the membrane expression of NMDA receptors, respectively (Yuen et al., 2005, 2008). Right, the NMDA receptor-dependent dendritic release is controlled by L-type calcium channels (Colgan et al., 2012), negatively by 5-HT_1A_ and positively by 5-HT_2B_ receptors at dendritic “puncta” independently of action potential (Colgan et al., 2012).

One of the best described model is the leech Retzius giant serotonergic neurons, in which low electrical stimulation (induced by a single action potential) causes the somatodendritic release of serotonin as evaluated by amperometry (Bruns et al., 2000). This release lasts several seconds following initial stimulation (Trueta et al., 2003), allowing serotonin to spread to several micrometers. The initial stimulation triggers the opening of L-type calcium channels (Trueta et al., 2003), the release of serotonin from few serotonin-containing vesicles, which then via 5-HT_2_-receptor activation produces a Ca^2+^ release from intracellular calcium stocks amplifying the release of serotonin from serotonin-containing vesicles (Trueta et al., 2004; Trueta and De-Miguel, 2012; Leon-Pinzon et al., 2014). In summary, somatodendritic release/exocytosis of serotonin occurs following low electrical stimulation and opening the L-type calcium channels. Ca^2+^-induced Ca^2+^ release is reinforced by activation of 5-HT_2_ receptors, which, by their coupling to the PLC pathway, amplify the serotonin release in a feed-forward manner (Leon-Pinzon et al., 2014; Figure 2). The resulting positive feedback loop maintains exocytosis for the following several seconds until the last vesicles in the cluster have fused (Trueta and De-Miguel, 2012; Leon-Pinzon et al., 2014). Taking into account the fact that some serotonergic neurons are capable of releasing glutamate, the co-release of this neuromodulator by simultaneous stimulation of the 5-HT_2_ receptors and NMDA receptors would induce a stronger signal and thus a rapid and strong reinforcement of serotonin transmission.

### In Rats

At somatodendritic level of dorsal raphe neurons, the presence of VMAT2 allows the accumulation of serotonin in vesicles (Chazal and Ralston, 1987). As shown by amperometry and 2-Photon calcium imaging, the non-synaptic somatodendritic release of serotonin-containing vesicles can be induced by the stimulation of calcium channels, or by activation of glutamatergic NMDA receptors in the absence of action potentials (de Kock et al., 2006). Using 3-Photon microscopy in living rat brain slices along with immunofluorescence and electron microscopy, vesicular serotonin release from soma and dendrites in the dorsal raphe was visualized for the first time (Kaushalya et al., 2008a; Colgan et al., 2012). These authors clearly established that punctate fluorescence does represent serotonin based on properties of multiphoton wavelength excitation, its detection in microdialysis serotonergic neurons, and its depletion upon exposure to serotonin synthesis inhibitors. Moreover, the presence of clusters of serotonin vesicles in dendrites was confirmed by (i) the immunolocalization of VMAT2 and the dendritic marker MAP2 with serotonin, (ii) the localization of VMAT2 vesicle clusters by electron microscopy in dendrites of serotonergic neuron, (iii) the size of dendritic serotonin/VMAT2 clusters comparable to the size of dendritic puncta and larger than terminal boutons, and (iv) the serotonin release from dendritic vesicles upon electrical stimulation or exposure to glutamate agonists, which requires extracellular Ca^2+^ and is blocked by the VMAT2 inhibitors. In the soma of serotonergic neurons, calcium channel- and NMDA receptor-activation by action potentials increases serotonin release (Figure 2-left); in proximal dendrites, both AMPA and NMDA receptor activation by back propagating action potentials may facilitate serotonin release; in contrast to standard release from axon terminals triggered by glutamate receptors, dendritic release of serotonin is independent of action potentials and requires L-type Ca^2+^ channels, but not sodium channels (Colgan et al., 2012; Figure 2-right).

Thus, unlike synaptic dendritic release in other spiking neurons, the dendritic release/exocytosis of serotonin is based on dendritic glutamatergic excitation without requirement for back-propagating action potentials, and is characterized by its sensitivity to NMDA, L-type Ca^2+^ channel blocker nimodipine. Furthermore, it was reported that upon electrical stimulation, the serotonin releasable pool is 300 times lower in comparison with dopamine despite comparable tissue content. Serotonin may be stored in vesicles or other compartments that do not exocytose consistent with a small quantity of serotonin available for release (Hashemi et al., 2012; Jennings, 2013). Hence, dorsal raphe dendrites release serotonin, and this function is physiologically and pharmacologically unique, although the molecular effectors and regulators of these dendritic non-synaptic events remain to be described in details.

## Serotonin Tone and Serotonergic Autoreceptors

### 5-HT_1_ Receptors

Neurotransmission by serotonin is tightly regulated by autoreceptors through negative feedback inhibition at somatodendritic levels (5-HT_1A_ receptors) of the raphe nuclei or at axonal levels (5-HT_1B_ receptors). The 5-HT_1A_ autoreceptor is found in the soma and dendrites of serotonergic neurons of raphe (Kia et al., 1996; Riad et al., 2000). In the raphe, the 5-HT_1A_ autoreceptor-mediated inhibition was for long time believed to be the only homeostatic feedback mechanism controlling the tonic firing rate, pacemaker-like, of serotonergic neurons, mainly based on *in vitro* data, for review see (Piñeyro and Blier, 1999; Vizi et al., 2010). However, accumulating results are weakening the traditional model postulating that serotonin neuron autoinhibition is mediated exclusively by the hyperpolarizing 5-HT_1A_ autoreceptor and that is the main factor controlling the pacemaker-like firing rate of serotonergic neurons, for review see (Andrade et al., 2015).

At somatodendritic levels, a reduction of expression of 5-HT_1A_ autoreceptors produces strong antidepressant effects, probably due to a reduction of the negative feedback on serotonergic neuron activity (Bortolozzi et al., 2012). Moreover, the genetic suppression of 5-HT_1A_ autoreceptors causes an anxiety-like behavior in the basal state, and a higher increase in serotonin release compared to wild-type mice in response to stress (Richardson-Jones et al., 2010). Deletion of either 5-HT_1A_ or 5-HT_1B_ autoreceptors (somatodendritic and axonal, respectively) does not modify brain serotonergic tone as assessed by microdialysis (Guilloux et al., 2011). Moreover, while complete deletion of both receptors in *Htr1a/1b^-/-^* mice affected the acute response to SSRIs in the forced swim test, the chronic effects of SSRIs were still observed in anxiety test (Guilloux et al., 2011). In mice with overexpression of 5-HT_1A_ autoreceptor, hypothermic response is increased, and both serotonin content and neuronal activity are decreased in the dorsal raphe. These mice display increased anxiety- and depression-like behaviors that are resistant to chronic antidepressant treatment (Vahid-Ansari et al., 2017). In addition, blockade of 5-HT_1A_ autoreceptors in dorsal raphe brain slices was found to have surprisingly no effect on the firing of the serotonergic neurons as reviewed in Liu et al. (2005). There is thus a discrepancy in 5-HT_1A_ receptors acting as a regulator of pace-maker homeostasis of serotonergic neurons between *in vivo* and *in vitro* studies.

Other studies showed that serotonergic cell groups can be interconnected, the dorsal raphe in particular receiving serotonergic inputs from the caudal raphe (Bang et al., 2012), which may implicate different types of serotonergic neurons. 5-HT_1A_ receptors participate in serotonergic neurons with different electrophysiological profiles, the inhibitory effect of 5-HT_1A_ receptors being superior in dorsal raphe than in median raphe neurons, suggesting greater negative feedback in the dorsal raphe (Beck et al., 2004). Similarly, Teissier et al. (2015) identified opposed consequences of dorsal vs. median raphe serotonergic neuron inhibition, suggesting that median raphe hyperactivity increases anxiety, whereas low dorsal over median raphe serotonergic activity ratio increases depression-like behavior. These observations suggest a heterogeneity of serotonergic neurons, which are interconnected but not necessarily located in the same serotonergic nucleus. It will thus be worth testing the effect of altering volume transmission in various raphe nuclei.

### 5-HT_2_ Receptors

On dorsal raphe slices, most serotonin neurons are hyperpolarized following the opening of GIRK channels by the application of a 5-HT_1A_ receptor agonist. In the presence of 5-HT_1A_-receptor antagonists, it has been reported that serotonin induces a depolarization, which can be blocked by different antagonists specific of Gq-coupled 5-HT_2_ receptors (Craven et al., 2001). In another study using rat brain slices, the stimulation of 5-HT_1A_ receptors also hyperpolarized most serotonin neurons, and about half of these neurons show also a depolarization in response to 5-HT_2_ receptor agonists (Marinelli et al., 2004). These data suggest that 5-HT_2_ receptors expressed by subsets of serotonergic neurons could participate in serotonin somatodendritic volume transmission. Local agonist stimulation of 5-HT_2B_ receptors in dorsal raphe increased extracellular serotonin, supporting an excitatory effect of this receptor on serotonergic neuron activity (Doly et al., 2008). Furthermore, a fraction of raphe serotonergic neurons coexpress both 5-HT_1A_ and 5-HT_2B_ receptors (Diaz et al., 2012). These observations confirmed that serotonergic neurons are heterogeneous by expressing different serotonin receptors and that both 5-HT_1A_ and 5-HT_2_ receptors could participate in serotonin tone regulation.

Putative positive regulation of dorsal raphe by 5-HT_2B_ receptors has been proposed (McDevitt and Neumaier, 2011). Strikingly, acute and long-term effects of SSRIs both in behavior and neurogenesis were eliminated after genetic ablation of 5-HT_2B_ receptors or upon selective antagonist treatment (Diaz et al., 2012). Conversely, pharmacological experiments indicated that acute agonist stimulation of 5-HT_2B_ receptors mimicked acute SSRI action (Diaz and Maroteaux, 2011) and that chronic agonist stimulation of 5-HT_2B_ receptors mimicked chronic SSRI action on behavior and neurogenesis, which were abolished in mice knocked-out (KO) for the 5-HT_2B_ receptor gene (*Htr2b^-^*^/^*^-^*) (Diaz et al., 2012). Accordingly, conditional KO mice for 5-HT_2B_ receptors only in serotonergic neurons (*Htr2b-cKO^5-HT^* mice), reproduced the lack of SSRI effects; these mice also displayed a reduced tonic firing frequency of dorsal raphe serotonin neurons, and a stronger hypothermic effect of 5-HT_1A_-autoreceptor stimulation (Belmer et al., 2018). The increased excitability of serotonergic neurons observed upon selective 5-HT_2B_-receptor overexpression in raphe serotonergic neurons confirmed the cell autonomous effect of this receptor. The excess of inhibitory control exerted by 5-HT_1A_ receptors in *Htr2b-cKO^5-HT^* mice may thus explain the lack of response to chronic SSRI in these mice. Conversely, the raphe neurons from mice expressing reduced amount of 5-HT_1A_ receptors (5-HT_1A_-Low) are more likely to fire at higher rates than control mice, consistent with decreased autoinhibition (Richardson-Jones et al., 2010). In parallel, Philippe et al. (2018) showed that an increased 5-HT_1A_-autoreceptor binding and function led to reduced serotonergic tone, increased anxiety-depression-like behaviors, and induced mice to be resistant to chronic fluoxetine. A higher 5-HT_1A_-autoreceptor reactivity and a lower firing activity of these neurons was observed in *Htr2b-cKO^5-HT^* mice (Belmer et al., 2018). Confirmation of these findings have been obtained in mice expressing the activator Gq-coupled DREADDS hM3Dq (similar to 5-HT_2B_ receptor’s coupling) in serotonergic neurons, which demonstrates, upon stimulation, an increase in serotonergic neurons firing rates (Teissier et al., 2015) and an antidepressant-like behavioral response (Urban et al., 2016). On the contrary, mice expressing the inhibitory Gi-coupled DREADDS hM4Di (similar to 5-HT_1A_ receptor’s coupling) in serotonergic neurons display, upon stimulation, a decrease in serotonin neuronal firing rates (Teissier et al., 2015). The serotonergic tone may thus result from the opposite control exerted by cross-regulation between Gi-coupled 5-HT_1A_ and Gq-coupled 5-HT_2B_ receptors on serotonergic neurons (Belmer and Maroteaux, 2018).

Interestingly, frog motor neurons showed potentiation of NMDA-induced depolarization by serotonin. The underlying mechanism involves: (1) activation of 5-HT_2B_ receptors; (2) activation of a Gq-protein; (3) a transduction mechanism causing an influx of extracellular Ca^2+^ through L-type calcium channels; (4) binding of Ca^2+^ to calmodulin; and (5) reduction of the open-channel block of the NMDA receptor produced by physiological concentration of Mg^2+^ ions (Holohean and Hackman, 2004). Furthermore, Bigford et al. (2012), showed that either 5-HT_2B_ or 5-HT_2C_ receptor antiserum immunoprecipitated GluN1 subunit of NMDA receptors, suggesting that these receptor subtypes are able to interact in complexes with NMDA receptors and our unpublished data confirmed a 5-HT_2B_- and GluN1-receptor association. Independently, the 5-HT_2B_ receptor, which is expressed in stomach and cardiomyocytes, has been reported to act via L-type calcium channels in both tissues (Cox and Cohen, 1996; Bai et al., 2010) and activation of 5-HT_2B_ receptors triggered also intracellular calcium release from ryanodine-sensitive stores as shown in the leech somatodendritic release of serotonin (Leon-Pinzon et al., 2014). Together, these data indicate that somatodendritic release of serotonin is a model in which 5-HT_2B_ receptors could participate and regulate the excitability of serotonergic neurons together with 5-HT_1A_ receptors.

The mechanism by which these two receptors interact remains to be described as well as the associated partners and intracellular pathways involved in the regulation of serotonergic tone at the level of serotonin neurons themselves.

## Volume Transmission, SERT, and SSRI Antidepressants

The serotonin transporter SERT by regulating extracellular levels of serotonin is a major partner in the regulation of serotonin tone (Piñeyro and Blier, 1999; Vizi et al., 2010). Under normal conditions, evoked extracellular serotonin concentration shows strong firing frequency-dependence. Mice lacking SERT (KO mice) or treated with SSRIs display extracellular serotonin concentrations evoked by stimulation that tend to similar high levels at all frequencies, while in SERT overexpressing mice, evoked extracellular serotonin concentrations tend to equal low levels (Jennings et al., 2010). These findings, therefore, indicate that SERT plays a role of a frequency pass filter in regulating extracellular serotonin concentrations evoked by stimulation. The role of SERT in setting basal extracellular serotonin concentrations and detailed contribution to serotonergic tonic and volume transmission has yet to be investigated *in vivo*.

The therapeutic effects of SSRIs are initially triggered by blocking SERT. Microdialysis experiments have shown that acute SSRI injections increase extracellular levels of serotonin by approximately 400% in the dorsal raphe and nearly 200% in forebrain terminal regions (Invernizzi et al., 1992, 1997). The SSRI-dependent increases in extracellular serotonin concentration require Ca^2+^-dependent vesicular release, which should induce somatodendritic 5-HT_1A_ autoreceptor-mediated decreases in spontaneous release of serotonergic neurons (Gartside et al., 1995; Hajos et al., 1995). Following SSRI injection, although basal serotonergic firing rates should decrease, the tonic activity increases extracellular serotonin levels (Dankoski et al., 2016). Interestingly, local infusion in the dorsal raphe of a 5-HT_2B_ receptor agonist through the microdialysis probe produced an increase in extracellular serotonin concentration that could be blocked by 5-HT_2B_ receptor antagonist (Doly et al., 2008) and mimicked the SSRIs effects. These data support a contribution of this receptor subtype in carrier-dependent serotonin accumulation.

One mechanism by which SERT can contribute to the enhancement of extracellular serotonin includes reversed transport, i.e., by carrier-mediated efflux (Forrest et al., 2008; Sitte and Freissmuth, 2015). The “club drug” 3,4-methylenedioxy methamphetamine (MDMA, ecstasy) binds preferentially to and reverses the activity of SERT, by causing release of serotonin from vesicles. Acute pharmacological inhibition or genetic ablation of 5-HT_2B_ receptors in KO mice completely abolished MDMA-induced hyperlocomotion, sensitization, and serotonin release. Furthermore, the 5-HT_2B_ receptor dependence of MDMA-stimulated release of endogenous serotonin relies on its expression in serotonergic neurons as recently demonstrated in mice lacking 5-HT_2B_ receptors only in serotonergic neurons (*Htr2b-cKO^5-HT^* mice) (Belmer et al., 2018). These data support also a contribution of this receptor subtype in carrier-dependent serotonin efflux. Unlike serotonin release in soma or terminals, dendritic serotonin release in response to AMPA or NMDA receptor stimulation requires L-type Ca^2+^ channels. AMPA-evoked serotonin release measured with varying fluoxetine concentrations showed that somatic serotonin release has fivefold greater sensitivity to fluoxetine than responses from dendritic puncta (Colgan et al., 2012). Differences in SERT regulation, localization and/or function may explain this difference, since SERT immunoreactivity has been mainly found at the plasma membrane in extrasynaptic location including axonal varicosities, whereas in soma and dendrites it was mainly observed intracellularly (Vizi et al., 2010; Belmer et al., 2017).

The therapeutic effects induced by SSRIs rely on long-term neuroadaptations. Since the activation of 5-HT_1A_ autoreceptor decreases the activity of serotonin neurons (Commons, 2008), more than 2 weeks of SSRI treatment are necessary to observe a decreased expression of 5-HT_1A_ receptors in serotonergic neurons (Popa et al., 2010). This decrease in expression of 5-HT_1A_ receptors, which is followed by an increase in the firing of serotonergic neurons, has been proposed to explain the clinical delay of the antidepressant effect of SSRIs (Adell et al., 2002; Santarelli et al., 2003; Richardson-Jones et al., 2010; Rainer et al., 2012). In SERT KO mice, 5-HT_1A_ autoreceptors are desensitized in raphe nuclei, while they remain intact in post-synaptic neurons (Fabre et al., 2000). This desensitization of 5-HT_1A_ autoreceptors in the raphe is thought to be due to the chronic accumulation of extracellular serotonin in the absence of uptake (Soiza-Reilly et al., 2015). The lack of acute and chronic SSRI efficacy observed in *Htr2b-cKO^5-HT^* mice is associated with a reduced tonic firing frequency of dorsal raphe serotonin neurons, whereas the selective 5-HT_2B_-receptor overexpression in raphe serotonergic neurons increases the excitability of these neurons (Belmer et al., 2018). Together with the observation that agonist stimulation of 5-HT_2B_ receptors is sufficient to reproduce SSRI effects including raphe serotonin accumulation, these results support that the reduction in 5-HT_1A_ receptor activity drives the antidepressant efficacy that may involve SERT regulation.

Colgan et al. (2012) proposed that the differential regulation between somatic vs. dendritic serotonin release may explain the antidepressant effects of inhibitors of NMDA receptors like ketamine (Machado-Vieira et al., 2009; Casamassima et al., 2010). Ketamine, has recently been shown to increase serotonin in prefrontal cortex, which correlates with antidepressant-like activity in the forced swimming test; its antidepressant-like activity requires activation of raphe AMPA receptors that recruits the prefrontal cortex neural circuit (Pham et al., 2017). Furthermore, AMPA receptor-dependent serotonin release and subsequent 5-HT_1A_ receptor stimulation may be involved in the actions of an mGlu2/3 receptor antagonist and ketamine in the NSF test (Fukumoto et al., 2014). However, it has been reported that a direct activation of AMPA receptors by ketamine metabolites and mTOR signaling is sufficient to increase synaptogenesis in prefrontal cortical pyramidal neurons and to enhance serotonergic neurotransmission via descending inputs to the raphe nuclei or even by a direct inhibition of NMDA receptors localized on GABAergic interneurons, for reviews see (Artigas et al., 2018; Zanos and Gould, 2018). It is therefore unlikely that the rapid antidepressant effects of NMDA receptor inhibitors act through a control of serotonergic tone, which would require time to be efficient, but through a direct control of upstream targets.

## Genetic Variants of Molecules Putatively Associated to Volume Transmission

Interestingly, human polymorphisms associated to psychiatric diseases have been found in genes encoding molecules putatively involved in somatodendritic release, including voltage-gated L-type calcium channel subunit, 5-HT_2B_ receptor, 5-HT_1A_ receptor, VMAT-2, or SERT. Single-nucleotide polymorphisms (SNPs) in the α1 subunit (*CACNA1C*) of the L-type calcium channels Cav1.2 rank among the most consistent and replicable genetics findings in psychiatry and have been associated with schizophrenia, bipolar disorder and major depression (Casamassima et al., 2010; Dedic et al., 2018) and more recently with treatment resistant depression (Fabbri et al., 2018). In humans, a loss-of-function SNP of 5-HT_2B_ receptors is associated with serotonin-dependent phenotypes, including impulsivity and suicidality (Bevilacqua et al., 2010). Association studies with the functional 5-HT_1A_ receptor promoter SNP rs6295 showed that patients present early deficits in cognitive, fear and stress reactivity that may lead to depression (Albert and Fiori, 2014). A specific haplotype in SLC18A2, the gene encoding VMAT-2, was significantly associated with depression symptoms in men (Christiansen et al., 2007). Furthermore, a significant association was found between post-traumatic stress disorder (diagnosis) and SNPs in SLC18A2 (Solovieff et al., 2014). Carriers of the short allele of the promoter polymorphism of SERT gene (5-HTTPR) have increased anxiety-related traits and elevated risk of depression (Pezawas et al., 2005). Evidence points to a lower response to SSRIs among Caucasian patients with the 5-HTTPR short genotype and among (Asian) patients with the STin2 10/12 genotype (Smits et al., 2008). However, humans carrying the short variant of the 5-HTTPR outperform subjects carrying the long allele in an array of cognitive and social tasks (Homberg and Lesch, 2011). So, one has to be careful in interpreting data from human gene polymorphism, without extensive characterization of their physiological consequences. These human polymorphisms that are associated to psychiatric diseases have then to be validated in models of serotonin somatodendritic release.

## Conclusion

Our understanding of serotonin transmission has been limited by technical problems. This review has summarized different mode of serotonin transmission and how they could impact behavioral and antidepressant efficacy. A better description of the molecular mechanisms involved in regulating serotonin somatodendritic release *in vivo*, using for example 3-Photons microscopy, is necessary to identify the impact of various modes of serotonin release and to unravel the mechanisms of tonic serotonin level regulation. These data should indicate if different modes of serotonin release mediate distinct behavioral effects. Understanding whether and how serotonin tone is controlled may also increase our understanding how its impact on behavior. By deciphering the molecular mechanisms of serotonin release that regulate firing patterns we should be able to increase our knowledge of serotonin function in physiological and pathophysiological situations. This should ultimately allow us to improve treatment of psychiatric disorders involving serotonin, such as depression.

## Author Contributions

All authors collected references, wrote the manuscript, and prepared the figures.

## Conflict of Interest Statement

The authors declare that the research was conducted in the absence of any commercial or financial relationships that could be construed as a potential conflict of interest.
